# Development of a Balloon Tamponade Task Trainer

**DOI:** 10.7759/cureus.21343

**Published:** 2022-01-17

**Authors:** Christopher E San Miguel, Kimberly Bambach, David P Way, Scott Winfield, Jennifer Yee

**Affiliations:** 1 Department of Emergency Medicine, The Ohio State University College of Medicine, Columbus, USA; 2 Clinical Skills Education and Assessment Center, The Ohio State University College of Medicine, Columbus, USA

**Keywords:** task trainer development, balloon tamponade, esophageal and gastric varices, hemorrhage control, gastroenterology, critical care, emergency medicine, medical simulation

## Abstract

Variceal hemorrhage is a life-threatening complication of patients with cirrhosis. If a patient is hemodynamically unstable and unable to undergo endoscopic therapy, a balloon tamponade device may be placed to temporize the hemorrhage until definitive management may be performed. Placement of these devices may be performed by practitioners of several different medical specialties. Placement of balloon tamponade devices requires multiple steps and several different pieces of equipment. Performing the procedure incorrectly can lead to iatrogenic injuries such as esophageal necrosis or perforation. Since this is a relatively rare procedure often placed under high-stress situations, practicing in a low-stakes setting, such as a simulation lab, allows practitioners to hone their skills. Commercially available task trainers for balloon tamponade device placement are not available. In this paper, we describe how to modify an inexpensive airway task trainer for this purpose using commonly available and cost-effective materials.

## Introduction

Acute variceal bleeding is a known complication for patients with cirrhosis, associated with a mortality rate of 10-20% [1]. If patients are too hemodynamically unstable to undergo definitive treatment, a balloon tamponade device may be placed to temporize the hemorrhage while ongoing resuscitative measures may be simultaneously performed, such as administration of blood products. Placement of these devices may be placed by practitioners of multiple medical specialties, including emergency medicine, internal medicine, critical care, and gastroenterology.

Placement of a balloon tamponade device requires gathering multiple pieces of equipment and timely execution of multiple steps in the correct order to avoid iatrogenic complications, such as esophageal necrosis [2], esophageal rupture [3-6], tracheal rupture [7-8], asphyxiation [2], endobronchial malpositioning [9], and upper airway obstruction due to tube migration [10]. This scenario often occurs in emergent high-stress settings with ongoing simultaneous resuscitation of hemorrhagic shock. Simulation lends itself well to reviewing high-risk, low-frequency procedures in a psychologically safe environment. Deliberate practice of psychomotor skills to meet a predetermined standard, a component of mastery-based learning, has demonstrated not only better procedural performance [11-13] but also improved patient outcomes [14-16].

Mastery-based learning is supported by practicing individual procedural steps until mastery is achieved. Realistic haptic, visual, and environmental feedback that is specific to the critical tasks and behaviors required for successful procedural completion should be provided to the trainee. In order to practice this procedure with a degree of fidelity, a task trainer is required to simulate not only the initial placement of the balloon tamponade device but also inflation of both balloons, application of traction, and measurement of esophageal balloon pressure. Reviewing the entire procedure allows for the identification of the individual steps at highest risk for possible iatrogenic injuries, such as device malposition, inappropriate balloon inflation, or excessive device traction. A commercially available task trainer for balloon tamponade device placement is not currently available. Young et al. described a modified task trainer in 2017 [17], that was built using PVC piping, vinyl tubing and a soda bottle with each component seated firmly within the next. Their rigid simulated stomach that only allowed for feedback in the form of visualization of initial device placement, but did not allow for either balloon to be fully inflated. Specific materials were required to fit precisely within each other. Here, we describe the creation of a cost-effective modified task trainer that allows learners to engage in repetitive deliberate practice for the entire process of balloon tamponade placement using equipment that is commonly available in simulation centers.

## Technical report

Equipment used in the development of the modified task trainer for the study is listed in Table 1. Approximately 8 hours were used for initial task trainer development, with subsequent task trainer creation requiring 20 minutes for assembly.

**Table 1:** Equipment Used to Create Modified Task Trainer with Approximate Costs

Two zip ties	Less than $1.00 each
Two 15 inch-long pieces of Penrose	$2.00 each
One 30 mL syringe with the plunger removed	$2.00 each
One bulb syringe with the bulb cut off	$3.00 each
Silk tape	$3.00 each
Bag-valve-mask with pressure-relief valve removed	$13.00 each
One Laerdal® Airway Management Trainer with the rubber stomach removed	$2,349.00 each

To create the simulated esophagus, two 15-inch-long pieces of Penrose were cut longitudinally down their seam. Both cut edges of Penrose were wiped down with an alcohol swab then glued along the longitudinal edge of the second piece of Penrose by overlying the edges by approximately 1.5 cm, creating one large piece that had an unchanged 15-inch length.

The bulb and distal end of the bulb syringe were cut off and discarded. The Penrose was then passed in a distal to proximal fashion through the smooth edge of the proximal portion of the syringe. Once 2 cm of Penrose was extended beyond the lipped end of the syringe, the end of the Penrose was folded back onto the syringe lip and the Penrose edge was secured onto the syringe with tape. The Penrose-wrapped edge of the syringe then served as the lower esophageal sphincter which fit into the bag-valve-mask stomach. Once the distal tip of the esophageal tamponade device was placed, it allowed for both gastric and esophageal balloons to be fully inflated to their appropriate volume and pressure respectively, simulating the complete execution of the procedure. Additionally, this allowed for visualization of the balloons for training purposes.

The self-inflating bag of a bag-valve-mask served as the stomach. The pressure-relief valve was pulled off the bag and the Penrose-wrapped lip of the syringe was pushed into the resultant opening, forming the simulated gastroesophageal junction. A zip tie was used to secure the Penrose-wrapped adaptor around the outside of the end of the bag-valve-mask. On the other end of the self-inflating bag, the bag was cut around the distal end.

**Figure 1:**
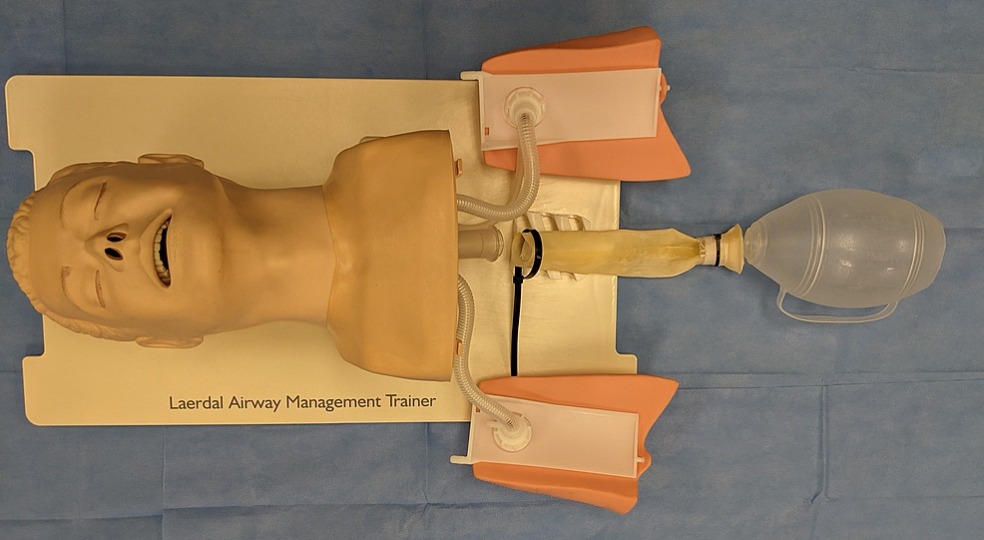
Attaching the Penrose Esophagus to the Airway Task Trainer

The stomach was removed from a Laerdal® Airway Management Trainer (Laerdal Medical Corporation, NY, USA), and a 30 mL syringe with the plunger removed was inserted into the mouth and was seated within the proximal esophagus. The free end of the Penrose was attached to the Laerdal® airway task trainer’s esophageal tubing and a zip tie was used to externally secure the proximal tubing to the airway task trainer (Figure 1).

**Figure 2:**
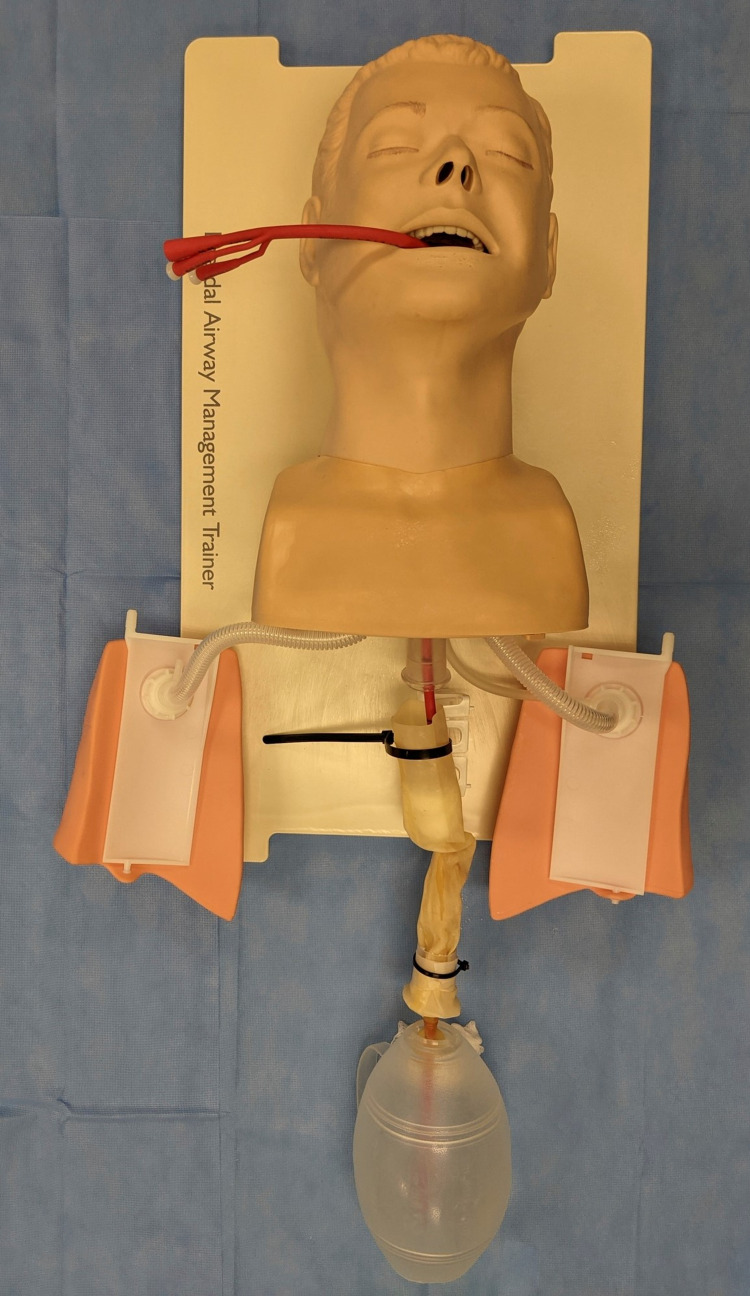
Final Product in Use with a Balloon Tamponade Device—Attachments Not Yet Secured

Lubrication spray was then liberally applied to the posterior oropharynx of the task trainer as well as the interior of the Penrose. The trainer was intubated prior to balloon tamponade placement to reflect the clinical practice of prioritizing airway management before device placement to prevent aspiration. The final product with a balloon tamponade device is depicted in Figure 2 (esophagus is not yet secured to better demonstrate attachment points).

## Discussion

This report describes a cost- and time-effective development of a modified task trainer for balloon tamponade placement, which does not currently exist in the commercial market. Technical expertise is not required for task trainer development and the materials used for development are available in many simulation centers. Using this task trainer, learners from various disciplines may practice preparing necessary equipment, placing the device within the esophagus and stomach, insufflating both balloons, and visualizing potential complications if balloons are improperly positioned when inflated. Modifications to the airway task trainer are not permanent, and other than cutting zip ties, can be reversed without further consumption of materials.

Studies have demonstrated that mastery-based learning not only benefits learners’ procedural skillsets [11-13] but also has a downstream effect on improved patient safety [14-16]. With mastery-based learning, realistic simulations must be available to learners so that they are able to engage in deliberate practice of procedures, using materials that they would use in the clinical setting and with task trainers that provide the haptic, visual, technical and environmental feedback required for attaining mastery.

This modified task trainer was prone to difficulty advancing the device down the esophagus into the stomach, which was mitigated by ensuring appropriate lubrication of both the Penrose and the balloon tamponade device prior to placement attempts. The facilitator occasionally had to help pull the distal end of the device through the stomach using Magill forceps. Depending on goals of the facilitator, the task trainer may represent limited fidelity as learners were able to visualize the depth of the balloon in real-time prior to radiographic confirmation of placement unless the task trainer was covered with a sheet from the neck down. Future developments may focus on adding the ability to simulate active bleeding.

## Conclusions

This cost-effective modified task trainer accurately depicts proximal airway and gastrointestinal anatomy required to master placement of a balloon tamponade device. We have described a task trainer with a semi-rigid stomach which allows for full insufflation of both the gastric and esophageal balloons, therefore increasing fidelity of simulating device placement. Prevention of iatrogenic injuries may be reviewed by visually demonstrating the consequences of incorrect balloon placement. Learners are able to practice this low-frequency, multistep procedure in a psychologically safe environment using this task trainer.
